# Comparison of mini-open repair system and percutaneous repair for acute Achilles tendon rupture

**DOI:** 10.1186/s12891-021-04802-8

**Published:** 2021-10-30

**Authors:** Yong Li, Qiang Jiang, Hua Chen, Hongkui Xin, Qing He, Dike Ruan

**Affiliations:** 1 Department of Orthopedics Surgery, The 6th Medical Center of Chinese PLA General Hospital, No. 6, Fucheng Road, Haidian District, Beijing, 100048 People’s Republic of China; 2 Department of Orthopedics, Chinese PLA General Hospital, No. 28 Fuxing Road, Beijing, 100048 People’s Republic of China

**Keywords:** Acute Achilles tendon rupture, Minimally invasive, Percutaneous, Mini-open

## Abstract

**Background:**

To reduce incision complications, minimally invasive operative approaches for treatment with acute Achilles tendon rupture have been developed, such as Mini-open repair and percutaneous repair. Which technique is the better surgical option? In the present study, we compared the two surgical procedures— modified Mini-open repair versus percutaneous repair—in the treatment of acute Achilles tendon rupture.

**Methods:**

From January 2016 to November 2018, 68 matched patients with acute Achilles tendon rupture were divided into treatment group (Mini-open with modified Ma-Griffith technique) and control group (the Ma–Griffith technique). The patients were then treated with different surgical techniques and followed up for no less than 24 months, and the functional outcome scores and complications were retrospectively evaluated.

**Results:**

The mean follow-up time in Mini-open repair group was 29.0±2.9 months, and that in control group was 27.9±2.9 months (*P*=0.147). The Mini-open repair group showed reliably higher American Orthopedic Foot and Ankle Society (AOFAS) Ankle-Hindfoot Score and Achilles tendon Total Rupture Score (ATRS) than the control group in functional assessment (95.0±3.8 vs. 92.3±5.3, *P*=0.000; 93.8±3.8 vs. 90.9±4.5,*P*=0.000). There was no cases of sural nerve injury in Mini-open repair group, whereas the percutaneous repair group had 5 cases of the same (*P*=0.027). No significant differences were found in the calf circumference (32.3±3.9 vs. 31.8±3.6) (*P*=0.564), range of motion of the ankle (51.3±4.8 vs. 50.5±4.2, *P*=0.362), or wound complications (34/0 vs. 34/0) (*P*=1.000) between the two groups at the end of the follow-up time. However, the percutaneous repair group had a shorter average operating time (23.1±5.2 min) than that of the Mini-open repair group (27.7±4.3 min) (*P*=0.000).

**Conclusions:**

Acute Achilles tendon ruptures may be treated successfully with a new Mini-open repair system or percutaneous repair technique. However, the Mini-open repair system may represent a superior surgical option, since it offers advantages in terms of direct visual control of the repair, AOFAS Ankle-Hindfoot Score, Achilles tendon Total Rupture Score and risk of sural nerve palsy.

**Study design:**

Case-control studies, Level of evidence, 3.

**Supplementary Information:**

The online version contains supplementary material available at 10.1186/s12891-021-04802-8.

## Introduction

Acute rupture of the Achilles tendon is one of the most common types of tendon ruptures in the human body [1]. This type of rupture commonly occurs at the location of the tendon with poor blood supply—that is, 2 cm to 6 cm above the insertion site. Because of the imperfections of surgical techniques [2, 3] and close relationship with the paratenon and plantar fascia [4], the optimal treatment of acute Achilles tendon ruptures is still under debate [5].

To reduce incision complications, minimally invasive operative approaches have been developed, such as percutaneous repair and Mini-open repair. Percutaneous suture technique is widely used by many surgeons in Achilles tendon repair, but sural nerve injury remains a problem. Sural nerve entrapment is one of the most common complications after percutaneous surgery [6–9]. The careful placement of stab incisions to expose the nerve so as to avoid it has been advocated. In addition, in order to reduce the risk of sural nerve injury, some surgeons use curved ring forceps [10, 11] or shaping Kirschner wires [12] for assistance, but it remains a challenge to prevent the sural nerve from being punctured or entrapment. In 2019, Carmont and Maffulli reported the results about percutaneous Bunnel type repairs for the treatment of acute Achilles tendon ruptures [13]. The rate of sural nerve damage remains as high as 6.8%. To reduce incision complications and nerve damage, various limited-open repair techniques have been developed recently [14–18]. According to the Kakiuchi’s suture method [12], Assal et al developed a device, later known as the Achillon® System™, and they published a prospective review of 87 patients treated for acute Achilles tendon rupture using this device in 2002 [19]. The invention of Achillon is a step forward in Mini-open treatment of the Achilles tendon. A meta-analysis [20] reported fewer wound complications with the Achillon device and no differences in rerupture rate, sural nerve injury, return to sports, or American Orthopaedic Foot and Ankle Society (AOFAS) score compared with open repair. Although the design of the Achillon device is ingenious, one of the disadvantages of Achillon device is that suture crossing is cumbersome, and the crossing sutures may cut through the Achilles tendon [21]. It affects the tensile strength of the Achilles tendon after repair. This device requires at least 6 sutures, and there should be at least 6 knots at the broken end. As a result, it would increase suture reactivity, which can affect postoperative recovery and Achilles tendon function. In 2010, the another Mini-open Repair System (PARS, Arthrex, Inc, Naples, FL) has been available. This device is similar to the Achillon device, but includes nonlocking and locking sutures to better grasp the tendon ends and potentially improve the strength of the repair. Although the PARS reduces the complications related to wounds and sural nerve entrapment, it is still relatively complex in the procedure, and also requires longer operating time.

**Fig. 1:**
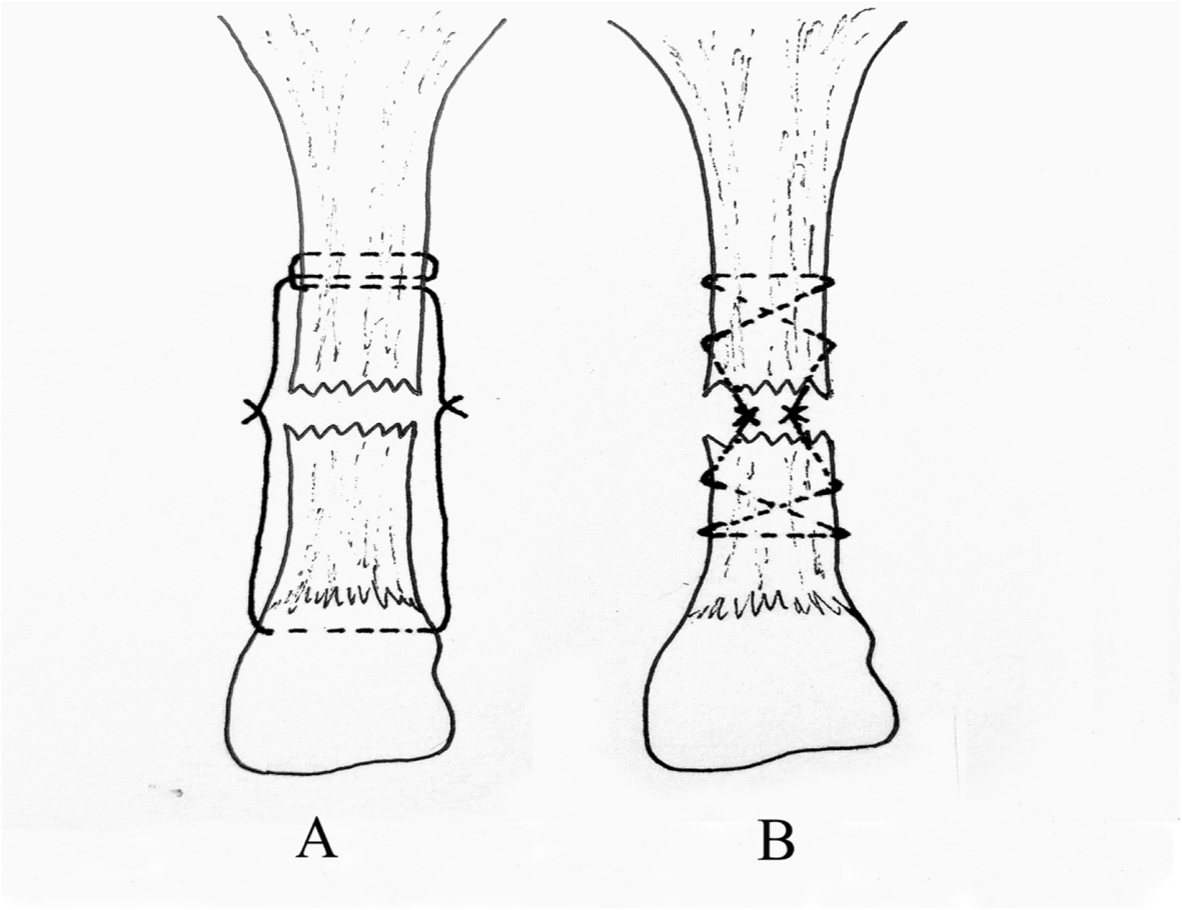
The principle of two kinds of minimally invasive anastomosis of Achilles tendon. **A** Mini-open repair system. **B** Percutaneous minimally invasive anastomosis (Ma and Griffith’s technique)

Which technique is the better surgical option for treatment with acute Achilles tendon rupture? In the present study, we used a new Mini-Open Achilles tendon repair system with modified Ma-Griffith technique (Fig. 1) [22, 23]. This suture system was based on the Bunnell suture method, which was different from the Achillon system. This new device requires at least 2 sutures and 2 knots at the broken end. In this retrospective control-matched study, we compared the two surgical procedures—Mini-open repair versus percutaneous repair—in the treatment of acute Achilles tendon rupture.

### Patients and methods

This control-matched study was conducted at the Navy General Hospital of PLA. Using the database and the medical records (between January 2016 and November 2018), 34 patients with acute Achilles tendon rupture treated with Mini-open repair were matched to 34 patients treated with percutaneous minimally invasive anastomosis. The age(±5), sex, and BMI(±5) were similar and well-matched in both groups. This study approved by the ethical committee of the Navy General Hospital of PLA in 2016. All experiments were performed in accordance with relevant guidelines and regulations. Both the surgical interventions described in study were implemented as standard-of-care at hospital. The patients can withdraw from the study at any time without discrimination or retaliation, and the medical treatment and rights and interests will not be affected. Regardless of any patient who refuses to participate in the study, he/she will still receive appropriate surgical treatment. Sample-size estimation was based on what was needed to detect the difference in complications in the groups. We estimated that more than 30 patients in each group were enough to detect a 20% difference in AOFAS score or ATRS between groups, with the alpha set at 0.05 and beta at 0.1. An additional 10 % of total participants was planned for each group to make up for possible loss. All patients read the detailed information sheet and signed a written consent form.

The criteria for inclusion in this study were as follows: (1) Patients with acute, closed Achilles tendon rupture; (2) A positive Thompson test; (3) Presence of pitting as assessed by observation and palpation between the two broken ends of the Achilles tendon; and (4) Complete rupture of the Achilles tendon as observed by ultrasonic examination. By contrast, patients with incomplete rupture of the Achilles tendon or open injury, patients with a repair time of more than 2 weeks, and patients with incomplete clinical data were excluded.

### Percutaneous repair (the Ma–Griffith technique)

Percutaneous Achilles tendon repair was performed in this study in accordance with the Ma–Griffith technique (Fig. 1). The patient was placed in a prone position, and a tourniquet was applied. The specific Ma–Griffith technique used in this study has been referred to in previous studies [24–26]. We incorporate the benefits of some new percutaneous repair approaches to minimize sural nerve damage.

### Mini-open repair group

**Fig. 2:**
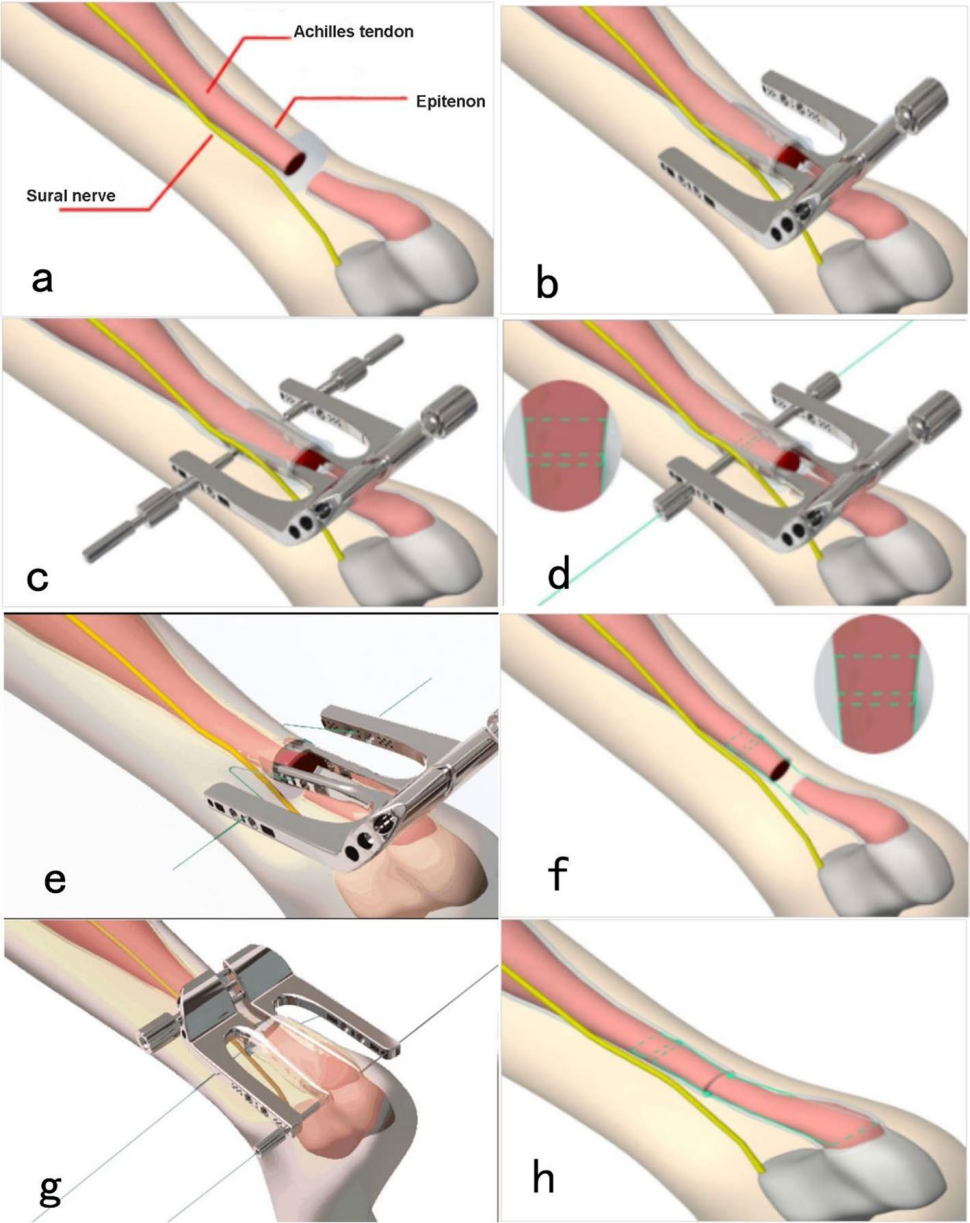
Operative approaches with Mini-open repair system. **a** The position relationship between sural nerve and Achilles tendon. **B** Establishment of the proximal surgical channel. **c** The epitenon is cut and proximal pilot hole is formed. d: Suturing of the ruptured Achilles tendon (AT) proximally. **e** The proximal suture was pulled out. **f** The proximal Achilles tendon was sutured and sural nerve was avoided successfully. **g** Suturing of the ruptured Achilles tendon distally. **h** Anastomosis of the ruptured Achilles tendon (AT) distally and proximally

The process was as follows. (1) *Establishment of the surgical incision.* The patient was placed in the prone position, and epidural anesthesia was applied. The end of the Achilles tendon was subsequently exposed by making an approximately 2–3 cm transverse incision at the level of tendon rupture (Fig. 2a). The proximal end of the Achilles tendon was also clamped and pulled out with hemostatic forceps. The channel instrument was inserted into the epitenon of the Achilles tendon along the fibers of the Achilles tendon. The instrument was then repeatedly pushed and pulled to achieve blunt separation of the proximal Achilles tendon and fascia (Fig. 2b). A longitudinal skin incision measuring approximately 5 mm was made along the guide holes on both sides of the proximal end of the tendon (Fig. 2c). The subcutaneous tissue was bluntly separated with hemostatic forceps (to protect the sural nerve from damage). (2) *Establishment of the proximal suture channel*. Two-sided tapered sleeves and center guides were placed along the proximal guide hole, and the suture channel was established (Fig.2d). (3) *Suturing of the ruptured Achilles tendon proximally and distally*. The physician threaded the needle once along the center guide on both sides while pulling the hemostatic forceps distally. The physician then adjusted the orientation of the guide needle and threaded the needle again without pulling the hemostatic forceps distally. The channel instrument was subsequently withdrawn, and the proximal suture was pulled out (Fig. 2e). Suturing of the ruptured Achilles tendon was completed proximally (Fig. 2f). Moreover, suturing of the ruptured Achilles tendon was completed distally by using the same method (Fig. 2g) as that for proximal suturing. (4) *Anastomosis of the ruptured Achilles tendon distally and proximally*. Tension was placed on the two ends of the suture, which were knotted for fixation (Fig. 2h). The broken ends were sutured with absorbable Vicryl Suture 3-0 to strengthen the anastomosis of the broken ends. The incision was sutured successively, and the long leg was fixed in plaster. The operation was completed.

### Postoperative care and rehabilitation

1. Non–weight-bearing equinus cast in place (0–2 weeks after surgery). A cast in a 20° to 25 ° “equinus” position was applied after both procedures and a below the knee gravity equinus cast was applied for approximately 2 weeks.
2. “Walker boot” period/muscle strength recovery period (3–10 weeks after surgery). After 2 weeks, the below the knee cast was removed and the patient began to ambulate in a “walker boot”, range-of-motion movements for the ankle were practiced, the leg muscles were strengthened, and normal gait was gradually restored. During the next 3-4 weeks the angle of the “walker boot” was gradually changed to a neutral position. The “walker boot” period was maintained for at least 8 weeks.
3. Muscle strengthening period (10 weeks after surgery and beyond). Ten weeks after surgery, ankle flexibility and leg muscle strength were improved to increase the stability of the lower limbs and to gradually restore motor function. Attempts were made to achieve full movement at the ankle. The ATRS [27, 28] and AOFAS [29, 30] ankle–hindfoot scale score were used to evaluate the clinical outcome at the last follow-up.
4. The patients were followed up for no less than 24 months, and the functional outcome scores and complications were retrospectively evaluated. Functional evaluation was based on the clinical AOFAS score and ATRS along with other findings, such as the length of the scar, neurologic deficit, calf circumference, and range of motion of the ankle. Whether there was no deep vein thrombosis or sural nerve injury was based on color Doppler ultrasound and electromyography.

### Statistical analysis

SPSS 23.0 was used for statistical analysis. The two groups were compared with respect to sex, age, follow-up time, operating time, hospital stay, calf circumference, AOFAS score, ATRS score, number of wound complications, sural nerve injury, and ankle ROM. Statistical analysis was conducted by an independent statistician not directly involved in the study. The Paired Samples t-Test, the results of which were expressed as the mean and standard deviation (SD), was used for the quantitative data analysis with equal variance assumed between the two groups. The Chi-square test was used to assess the qualitative data between the two groups. A *P* value of less than 0.05 was considered significant.

## Results

The baseline information and demographics of both groups are listed in Table 1. A total of 68 patients were enrolled in the Mini-open repair group with an average age of 32.3±6.9 y (range, 21–42 y) and the percutaneous repair group with an average age of 30.5±7.1 y (range, 18–40 y).

**Table 1:** Baseline characteristics of both groups

Variable	Mini-open repair group	Percutaneous repair group	*P* value
Age (years)	32.3±6.9	30.5±7.1	0.253^a^
Gender(M/F)	31/3	31/3	1.000^b^
Side(L/R) BMI (kg/m^2^) Diabetes mellitus (%) Smoking (%) Alcohol use (%) Corticosteroids (%) Peripheral vascular disease (%)	19/15 24.3±2.7 5.9 32.4 55.9 5.9 8.8	18/16 23.5±3.4 11.8 29.4 50.0 8.8 11.8	1.000^b^ 0.302^a^ 0.673^c^ 0.793^b^ 0.627^b^ 1.000^c^ 1.000^c^

^a^ *P*-value as determined by the Paired Samples t-Test;

^b^ The Chi-square test was used for the comparison of rates;

^C^ Fisher’s exact test was used when one or more expected values are less than 5

The follow-up data were summarized, and functional results were evaluated in both groups (Table 2). All patients in both groups were available for follow-up, with a mean follow-up time of 29.0±2.9 months in group A and 27.9±2.9 months in group B (*P*=0.147). The Mini-open repair group showed reliably higher AOFAS Ankle-Hindfoot Score and ATRS than the control group in functional assessment (95.0±3.8 vs. 92.3±5.3, *P*=0.000; 93.8±3.8 vs. 90.9±4.5, *P*=0.000). There was no cases of sural nerve injury in Mini-open repair group, whereas the percutaneous repair group had 5 cases of the same (*P*=0.027). No significant differences were found in the calf circumference (32.3±3.9 vs. 31.8±3.6) (*P*=0.564), range of motion of the ankle (51.3±4.8 vs. 50.5±4.2, *P*=0.362), or the number of wound necrosis or infection (34/0 vs. 34/0) (*P*=1.000) between the two groups at the end of the follow-up time. However, the percutaneous repair group had a shorter average operating time (23.1±5.2 min), compared with the Mini-open repair group (27.7±4.3 min) (*P*=0.000). No cases of sural nerve injury in the Mini-open repair group were reported, but five such cases were found in the percutaneous repair group (*P*=0.027).

**Table 2:** Comparison of the main follow-up data for both groups of patients

Variable	Mini-open repair group	percutaneous repair group	*P* value
Average operating time(min)	27.7±4.3	23.1±5.2	0.000^a^
Follow-up time(months)	29.0±2.9	27.9±2.9	0.147^a^
Calf circumference	32.3±3.9	31.8±3.6	0.564^a^
Re-rupture (n)	0	0	1.000^b^
Palpable knot (n)	5	8	0.355^b^
Scar tissue adhesions (n)	0	2	0.493^b^
Wound necrosis (n)	0	0	1.000^b^
Superficial infection (n)	0	0	1.000^b^
Deep infection (n)	0	0	1.000^b^
AOFAS score	95.0±3.8	92.3±5.3	0.000^a^
ATRS score	93.8±3.8	90.9±4.5	0.000 ^a^
Sural nerve palsy (n)	0	5	0.027^c^
Ankle ROM(°)	51.3±4.8	50.5±4.2	0.362^a^

^a^ *P*-value as determined by the Paired Samples t-Test

^b^ The Fisher two-sided exact test was used for the comparison of rates

^c^ The Fisher one-sided exact test was used for the comparison of rates

Multivariate analysis was performed to analyze the relationship. The age, BMI, Operating time, hospital stay, ATRS and ROM of ankle joint were taken as independent variables, while AOFAS was taken as dependent variables for linear regression analysis. The results were shown in the Supplement Tables 1 and 2.

## Discussion

**Fig. 3:**
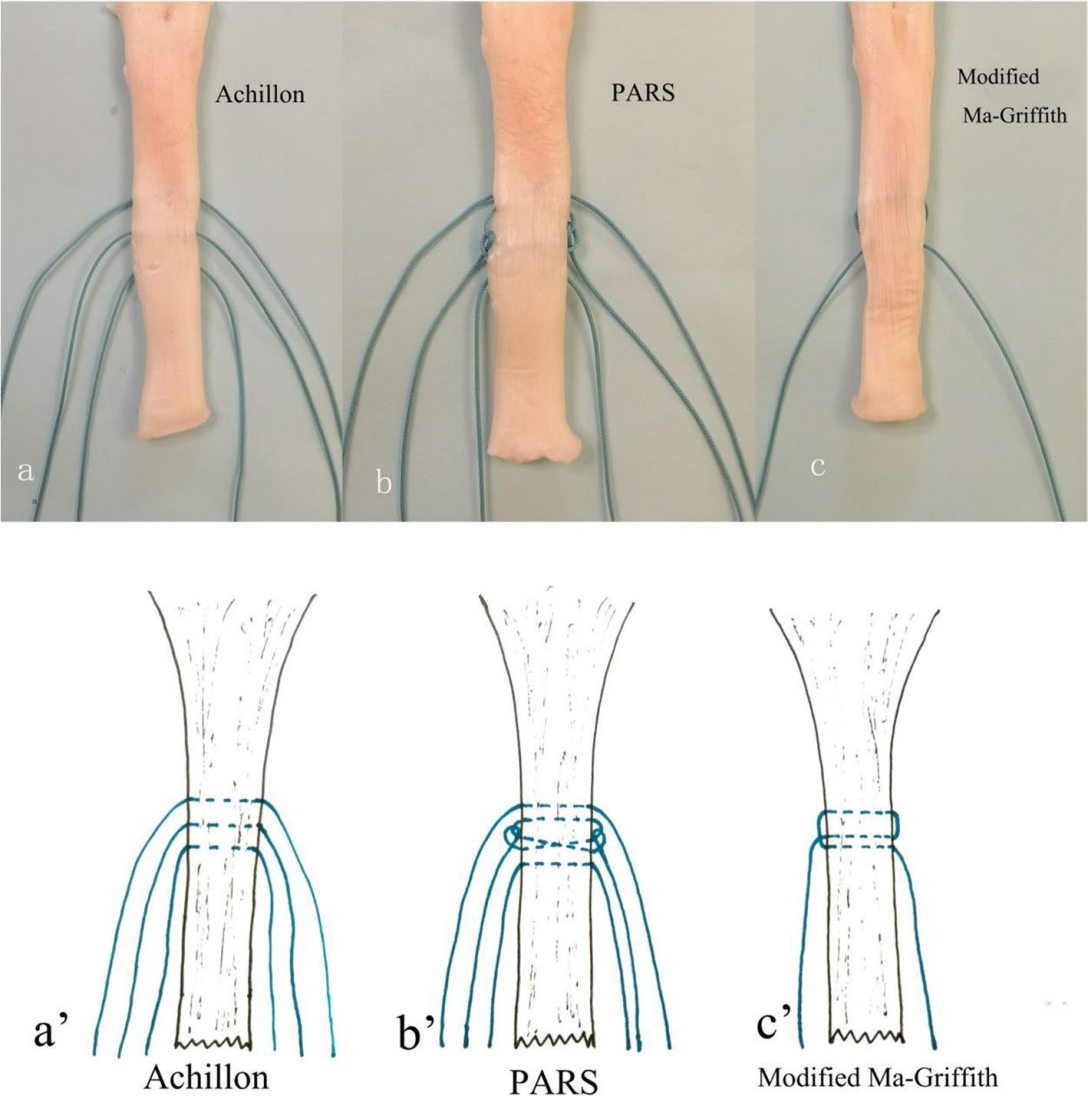
Photographs and simplified schematic diagrams illustrating the different repair constructs and suture configurations. **a,a’** Achillon repair, **b,b’** PARS repair, **c,c’** Mini-open repair

The choice of treatment for acute Achilles tendon rupture remains a challenge for surgeons. Despite hundreds of publications in the medical literature on the subject of acute rupture of the Achilles tendon, its optimal treatment remains under debate. One study from the Netherlands has described that although open repair (65%) was the most common surgical technique and Bunnell sutures (55%) were mostly applied, trauma surgeons and orthopaedic surgeons differed significantly on surgical technique (*p*= 0.001), suturing technique (*p*= 0.002) [31]. Surgical treatment can effectively reduce the rate of re-rupture and can lead to early functional recovery with exercise [32]. However, open surgery usually requires a long operative incision (average length of approximately 10 cm) and requires too much shedding of the Achilles tendon tissue, which can affect postoperative recovery [33]. Minimally invasive repair for Achilles tendon rupture has become widely applied to avoid long surgical incision, soft tissue necrosis, infection, and other related complications [34, 35]. Ma et al. introduced the use of percutaneous minimally invasive suture repair for Achilles tendon rupture [36]. Mini-open repair includes medial and lateral percutaneous–minimally invasive incisions and suture of the ruptured proximal tendon with a modified Bunnell suture and a diatal box suture [37]. Khan, R. J. et al. [6] concluded that compared with open surgical techniques, percutaneous techniques led to reductions in re-ruptures and overall complication rate. To better reconstruct the continuity of the tendon ends and reduce the risk of complications, the properties of the open and percutaneous techniques were combined [12]. An increasing number of orthopedic surgeons currently prefer to perform Mini-open procedures with surgical aid devices (Fig. 3) such as Tenolig [35, 38, 39], Achillon, PARS [34], or the Dresden instrument [19, 25, 40, 41] and at times integrate the method with ultrasound-guided approaches.

In the present study, we used a new minimally invasive and direct visual control of Achilles tendon suture system (Fig. 1). This Mini-open repair system was based on the Bunnell suture method, which was different from the Achillon system (Fig. 2). The proximal end of Achilles tendon is sutured with three transverse crosses of sutures by using an eccentric sleeve. We simplified the complex steps of Achillon and PARS repair and decreased the number of knots, facilitating the gliding of the Achilles tendon with the surrounding tissue after long-term repair and reducing the formation of keloids [42, 43]. So, this Mini-open repair system can reduce the risk of suture reactivity and make the broken end smoother. The aforementioned methods are the important factors affecting long-term functional recovery after Achilles tendon repair. In vitro studies showed no significant reduction in suture strength, although the number of stitches was decreased. After the proximal and distal sutures were tied, the strength of repair sufficiently met the requirements of early functional exercise. A study [23] performed a biomechanical comparison of the Mini-open repair repair system and three common Achilles tendon restoration techniques (Achillon, PARS, Krackow) in an *in vitro* model via a progressive rehabilitation program. Mini-open repair can achieve reliable suture strength with fewer stitches and knots, as strong as that of the open Krackow restoration, but weaker than those of the Achillon and PARS techniques. To a certain extent, the greater tensile strength of the suture used, the stronger the tensile strength of the Mini-open repair suture structure. Therefore, the repaired Achilles tendon exhibits high tensile strength, allows early functional exercise, and requires skills that can be easily mastered with a short learning curve. This Mini-open repair system has reduced suture knots, lowering the foreign body sensation of the suture knot and keloid after recovery and consequently improving the function and appearance of hindfoot. Our results suggest that both Mini-open repair and percutaneous repair can achieve satisfactory functional outcomes in patients with Achilles tendon. However, the Mini-open repair group showed reliably higher AOFAS Ankle-Hindfoot Score and ATRS than the control group in Function assessment (95.0±3.8 vs. 92.3±5.3, *P*=0.000; 93.8±3.8 vs. 90.9±4.5,*P*=0.000). The functional results are comparable to the results in several other investigations using open and mini-open repair techniques [44–47]. Calder et al. treated 25 patients by using an Achillon Achilles tendon suture device, and the follow-up AOFAS score was as high as 98.4 points [48]. Chen et al. performed Mini-open repair repair in 41 patients [22], and 90.5 was the reported AOFAS score 12 months after surgery. Multivariate analysis was performed to analyze the relationship. The results in the supplement tables showed that in the Mini-open repair group, the R^2^ value of in the model is 0.699 and it meant that the age, BMI, Operating time, hospital stay, ATRS and ROM of ankle joint could explain 69.9 % variations in AOFAS. The model was tested by the F-test (F=11.967, *P*<0.05), indicating that at least one of age, BMI, operating time, hospital stay, ankle range of motion and ATRS has an impact on the AOFAS Score. In the percutaneous repair group, the R^2^ value of model was 0.619 and it also passed the F-test (F=7.649, *P*<0.05), indicating that the age, BMI, a model could explain 61.9% of the variation in AOFAS.

Sural nerve palsy is one of the most important complications of minimally invasive repair of Achilles tendon rupture. A recent meta-analysis [49] suggests that sural nerve palsy is still a considerable complication of MIS. Initially, under the Ma–Griffith percutaneous technique, a sural nerve palsy rate reaching 60% has been reported. Haji et al. found that applying this technique [7] results in up to 10.5% transient sural nerve injury rate. Sutherland et al. treated 31 patients with this minimally invasive percutaneous suturing method, 5 of whom developed sural nerve injury [50]. In 2011, Taglialavoro et al. [51] reported that Tenolig group showed a lower risk of damage to the sural nerve compared to the Ma and Griffith technique(2/30 VS. 4/30). In the current study, the results of our classic percutaneous surgery were similar to those in other studies, with 5 cases of sural nerve injury. The sural nerve injury caused by this method may be mainly attributed to the nerve not being fully exposed during the operation and the suture needle being placed blindly, hence the risk of a direct puncture injury to the nerve. An increased risk of direct sural nerve injury or indirect irritation by sutures exists particularly when needles are pierced laterally into the proximal portion of the Achilles tendon. Simultaneously, it leads to tethering of the fascia cruris to the tendon. Therefore, minimally invasive Achilles tendon surgery should aim to avoid sural nerve injury. In this study, modified Mini-open repair was used to establish the suture channel so that the sural nerve was located outside the suture channel before suturing (Fig. 2), and the injury was effectively avoided during suture threading. In this study, no sural nerve injury occurred in the Mini-open repair group. On the basis of the results, Mini-open repair as a surgical option may be preferable to percutaneous repair for the treatment of acute Achilles tendon rupture because the former avoids damage to the sural nerves.

This study has certain limitations in control-matched designs. Selection bias was not avoided, considering that the surgical treatment to be performed was determined by the orthopedic surgeon. Although the two groups of patients were matched, only the sex, age and BMI of the patients were matched in the groups to maintain a sufficient number of patients.

## Conclusions

In conclusion, our results suggest that satisfactory functional outcomes can be obtained for both treatment methods. This new Mini-open repair system is easy to operate and the guide instrument of the system is placed deep into the paratenon, preventing the risk of a subcutaneous nerve being trapped in the suture itself. Mini-open repair may be the superior surgical option, given its advantages in terms of direct visual control of the repair, AOFAS Ankle-Hindfoot Score, ATRS and risk of sural nerve palsy.

## Supplementary Information

**Additional file 1: Supplement Table 1.** Multivariate analysis results in Mini-open repair group

## Data Availability

The datasets used and/or analysed during the current study are availablefrom the corresponding author on reasonable request.

## Abbreviations

AT: Achilles tendon
ATRS: Achilles tendon Total Rupture Score
AOFAS: American Orthopedic Foot and Ankle Society
SD: standard deviation
BMI: body mass index
