# Supplementary material for: Comparison of mini-open repair system and percutaneous repair for acute Achilles tendon rupture
Source: BMC Musculoskelet Disord. 2021 Oct 30;22:914. doi: 10.1186/s12891-021-04802-8 (PMC8556965; doi:10.1186/s12891-021-04802-8)
Supplement: Supplementary file 1 — Additional file 1: Supplement Table 1. Multivariate analysis results in Mini-open repair group [file 12891_2021_4802_MOESM1_ESM.docx]

[Supplement](javascript:;)  Table 1. Multivariate analysis results in Mini-open repair group

| variables | β | t Value | P value |
| --- | --- | --- | --- |
| Age | -0.170 | -1.473 | 0.153 |
| BMI | -0.378 | -2.956 | 0.007 |
| Operating time(min) | 0.131 | 0.772 | 0.447 |
| follow-up time | -0.043 | -0.240 | 0.812 |
| Hospital stay (days) | 0.087 | 0.849 | 0.404 |
| Ankle ROM | 0.069 | 0.676 | 0.505 |
| ATRS | 0.445 | 3.291 | 0.003 |

1. Dependent Variable: AOFAS
2. F=11.967, P<0.05
3. Adjusted R^2^=0.699

[Supplement](javascript:;)  Table 2. Multivariate analysis results in percutaneous repair group

| variables | β | t Value | P value |
| --- | --- | --- | --- |
| Age | -0.294 | -1.341 | 0.192 |
| BMI | -0.450 | -2.065 | 0.049 |
| Operating time(min) | 0.128 | 0.912 | 0.370 |
| follow-up time | -0.006 | -0.047 | 0.963 |
| Hospital stay (days) | 0.108 | 0.766 | 0.451 |
| Ankle ROM | 0.124 | 1.043 | 0.307 |
| ATRS | 0.163 | 0.880 | 0.387 |

1. Dependent Variable: AOFAS
2. F=7.649, P<0.05
3. Adjusted R^2^=0.619
